# New developments in imaging in ALS

**DOI:** 10.1007/s00415-025-13143-8

**Published:** 2025-05-12

**Authors:** Jana Kleinerova, Giorgia Querin, Pierre-Francois Pradat, We Fong Siah, Peter Bede

**Affiliations:** 1 Computational Neuroimaging Group (CNG), School of Medicine, Trinity College Dublin, Room 5.43, Pearse Street, Dublin 2, Dublin, Ireland; 2 Biomedical Imaging Laboratory, CNRS, INSERM, Sorbonne University, Paris, France; 3 Department of Neurology, Pitié-Salpêtrière University Hospital, Paris, France; 4 Department of Neurology, St James’s Hospital, Dublin, Ireland

**Keywords:** Amyotrophic lateral sclerosis, Motor neuron disease, Primary lateral sclerosis, Cerebellum, Frontotemporal dementia, Neuroimaging, Magnetic resonance imaging, Biomarkers

## Abstract

Neuroimaging in ALS has contributed considerable academic insights in recent years demonstrating genotype-specific topological changes decades before phenoconversion and characterising longitudinal propagation patterns in specific phenotypes. It has elucidated the radiological underpinnings of specific clinical phenomena such as pseudobulbar affect, apathy, behavioural change, spasticity, and language deficits. Academic concepts such as sexual dimorphism, motor reserve, cognitive reserve, adaptive changes, connectivity-based propagation, pathological stages, and compensatory mechanisms have also been evaluated by imaging. The underpinnings of extra-motor manifestations such as cerebellar, sensory, extrapyramidal and cognitive symptoms have been studied by purpose-designed imaging protocols. Clustering approaches have been implemented to uncover radiologically distinct disease subtypes and machine-learning models have been piloted to accurately classify individual patients into relevant diagnostic, phenotypic, and prognostic categories. Prediction models have been developed for survival in symptomatic patients and phenoconversion in asymptomatic mutation carriers. A range of novel imaging modalities have been implemented and 7 Tesla MRI platforms are increasingly being used in ALS studies. Non-ALS MND conditions, such as PLS, SBMA, and SMA, are now also being increasingly studied by quantitative neuroimaging approaches. A unifying theme of recent imaging papers is the departure from describing focal brain changes to focusing on dynamic structural and functional connectivity alterations. Progressive cortico-cortical, cortico-basal, cortico-cerebellar, cortico-bulbar, and cortico-spinal disconnection has been consistently demonstrated by recent studies and recognised as the primary driver of clinical decline. These studies have led the reconceptualisation of ALS as a “network” or “circuitry disease”.

## Introduction

Neuroimaging in amyotrophic lateral sclerosis (ALS) and other motor neuron diseases (MNDs) has contributed significant insights with regard to the preferential involvement of specific brain regions, phenotype-associated disease burden patterns, genotype-associated imaging signatures, longitudinal disease trajectories, and, more recently, early brain and spinal cord changes in asymptomatic mutation carriers [1, 2]. Despite this progress, translation of research findings into real-life clinical applications has been disappointingly slow. In this paper, we review some of the emerging trends in MND imaging, highlight the most important methodological advances, discuss some of the most striking conceptual shifts, examine the practical challenges associated with the development of clinical tools, and outline the most pressing research priorities going forward. Instead of adopting a systematic review format, we primarily focus on recent research papers signalling a paradigm shift from descriptive academic analyses to real-life clinical applications.

### Consensus imaging findings

**Fig. 1:**
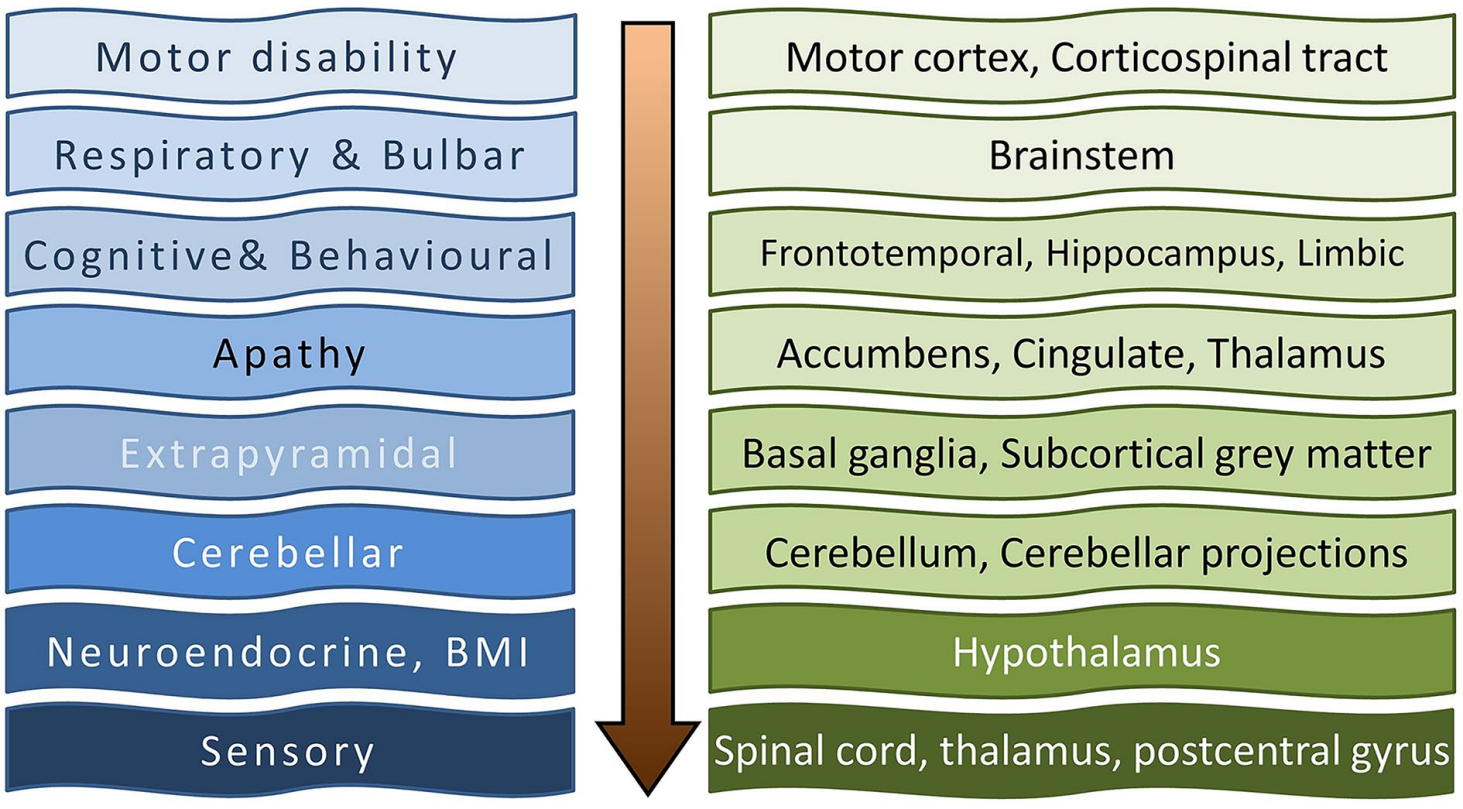
Expanding the clinical and anatomical spectrum of ALS from motor symptoms to extra-motor manifestations, *BMI* body mass index

**Fig. 2:**
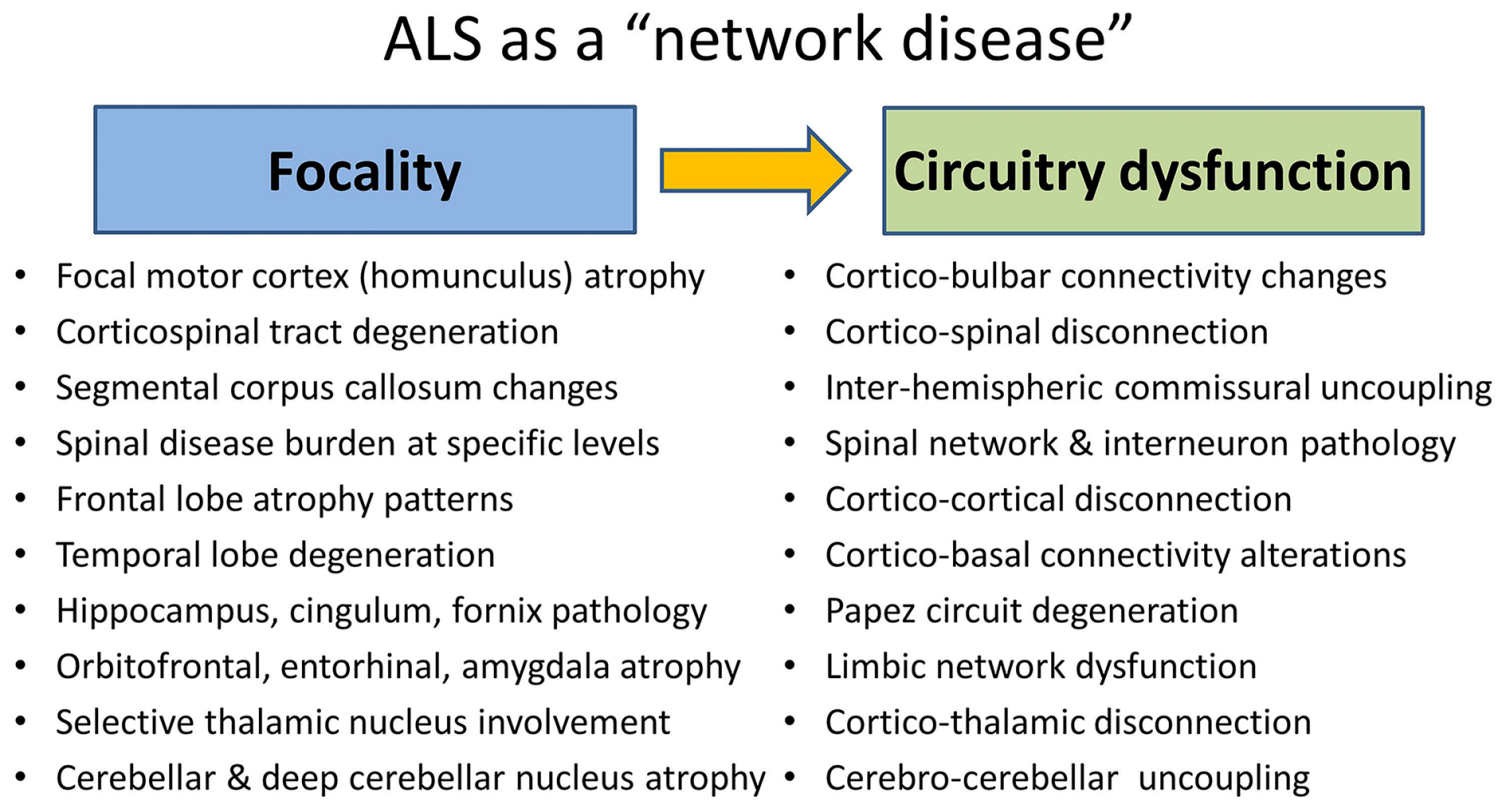
The reconceptualisation of ALS as a “network disease”: from the description of focal cerebral changes to the evaluation of circuitry dysfunction

Irrespective of the specific methodology utilised, nearly all robust imaging studies in ALS capture motor cortex, corticospinal tract, corpus callosum, and brainstem degeneration [3–6]. While the involvement of these regions is often regarded as pathognomonic of ALS, the same regions are also affected in PLS, HSP, and to some extent in other neurodegenerative disorders [7–9]. Early ALS imaging studies have primarily focused on the radiological correlates of motor disability and associations with motor phenotypes, whereas more recent studies have evaluated the underpinnings of extra-motor manifestations [10–14] (Fig. 1**).** With the recognition of neuropsychological deficits in ALS/MND, imaging studies have gradually started evaluating frontotemporal changes [15–17]. While initially extra-motor pathology was primarily associated with GGGGCC hexanucleotide repeat expansions in *C9orf72* [18, 19], more recent papers have clarified that significant frontotemporal atrophy and subcortical pathology in ALS are not unique to *C9orf72* carriers [20]. As clinical data indicates that there is a high incidence of cognitive, behavioural, and extrapyramidal manifestations in ALS, the imaging community has gradually turned their attention to the assessment of subcortical grey matter changes and the integrity of cerebral networks relayed by these structures [21–24]. While sporadically reported over the years, a relatively new frontier of ALS imaging is the assessment of cerebellar degeneration [25–27]. Cerebellar dysfunction is often exclusively linked to impaired coordination and poor balance, but cerebellar pathology also contributes to cognitive and behavioural manifestations, pseudobulbar affect, alterations of respiratory rhythm, changes in dexterity, impaired appetite regulation, and bulbar dysfunction [28–31]. Recent studies have not only confirmed significant intra-cerebellar disease burden, but also considerable cerebro-cerebellar disconnection [32]. This trend epitomises the evolution of imaging in ALS, namely that early studies focused primarily on the focal degeneration of specific brain structures, whereas more recent papers describe connectivity alterations between relevant brain regions [33], reconceptualising ALS as a network or circuitry disease (Fig. 2**)**. Connectivity in ALS has been evaluated by diverse techniques, diffusion imaging, resting-state functional MRI, EEG data, etc., but irrespective of the primary methodology, progressive disconnection has been captured as a consistent finding. As our clinical understanding of ALS has evolved from a relatively pure “motor-system” disease, to a “multi-system” disorder with frontotemporal [34], extrapyramidal [35, 36], and cerebellar manifestations [37], sensory dysfunction has also been gradually recognised [38, 39] (Fig. 1**.**). With the reconceptualisation of ALS as a multi-network, multi-region disease, imaging studies have slowly turned their attention to the radiological substrate of non-motor manifestations such as apathy, sensory dysfunction, disinhibition, pseudobulbar affect, deficits in social cognition, alterations in appetite, and so on [31, 40–44]. An interesting development in recent years is the targeted assessment of the hypothalamus and linking hypothalamic pathology to neuroendocrine manifestations, such as appetite regulation, weight, and metabolic profiles [45–47]. Another exciting frontier of ALS imaging is spinal cord imaging, which has gained considerable momentum in recent years [48, 49]. For decades, ALS imaging was disproportionately dominated by brain studies, and lower motor neuron components of the disease were strikingly underevaluated. This led to a relatively stereotyped oversight in imaging studies, namely that cerebral disease burden was often directly linked to motor disability overlooking the anterior horn (LMN) components of motor weakness. It is only relatively recently that the entire neuroaxis can be reliably imaged from the motor cortex to muscle. Quantitative spinal imaging has a number of methodological challenges, such as susceptibility to movement, respiratory, and cardiac effects; nonetheless, recent spinal studies readily detect lateral and posterior column degeneration in both symptomatic patients and asymptomatic mutation carriers [50, 51]. Spinal cord imaging in ALS has captured both the lower and upper motor neuron components of ALS pathophysiology and added important insights regarding sensory and respiratory involvement [52]. Muscle imaging in ALS is in its infancy, but important proof-of-concept papers have been published [53, 54]. Whole-body muscle mass reductions have been captured and the underpinnings of bulbar dysfunction also evaluated [55]. From a respiratory perspective, a series of innovative imaging studies have looked at the brainstem and the spinal and diaphragmatic components of respiratory compromise [56].

### Controversies and inconsistencies

Despite the considerable advances in the field of ALS imaging, some inconsistencies persist. Increased connectivity has been reported by multiple studies [57] between various brain regions which are sometimes interpreted as evidence of adaptive or compensatory processes [58]. Others interpret these findings as evidence of decreased inhibition. Nonetheless, increased connectivity detected by resting state fMRI studies are seldom supported by diffusion MRI data, which raises methodological questions regarding the validity of rsfMRI in late-stage ALS. Furthermore, while compensatory and adaptive changes have been suggested by multiple research groups [58], there is no compelling supporting evidence from post-mortem studies showing “hypertrophic” changes, increased synaptic density, or re-myelination. PET studies often show foci of hypermetabolism in various brain regions, especially in the cerebellum, but again, these are unlikely to represent compensatory change and more likely to be consistent with inflammatory change [59]. There are additional concerns about direct clinico-radiological correlations. Cognitive and behavioural changes are often solely attributed to frontotemporal degeneration, overlooking the possible contribution of cerebellar disease to these symptoms [60]. The laterality of radiological findings is relatively poorly characterised, handedness is not always taken into consideration, and often left and right hemispheric changes are averaged. Papers examining the radiological substrate of neuropsychological deficits often overlook the confounding effect of fatigue, apathy, hypoxia, and polypharmacy. The conceptual and methodological risks of direct clinico-radiological associations have been highlighted by a number of opinion papers [61]. Another pitfall of recent papers is the potential over-interpretation of presymptomatic findings. While cortical, white matter, and subcortical grey matter degenerations have been consistently identified in asymptomatic *SOD1* and *C9orf72* repeat expansion carriers [2, 62–64], these changes are likely to be specific to these genotypes and are unlikely to be representative of sporadic ALS. EEG is increasingly utilised in ALS due to its excellent temporal resolution and has confirmed phenotype-associated network alterations [65–67]. However, its practical drawbacks, such as the lack of infratentorial data acquired in most protocols and the indirect inferences on deep-brain function, are seldom acknowledged [68]. Similarly, while MEG is an excellent academic tool and has detected beta-band alterations in asymptomatic mutation carriers [69], its real-life biomarker potential is limited by the lack of its availability at most centres [68]. Compared to other neurodegenerative conditions, MRI is poorly tolerated by patients with ALS, and longitudinal ALS studies suffer from notoriously high attrition rates, hindering the accurate mapping of disease trajectories [70, 71]. Another contentious facet of ALS imaging studies is the choice of control participants. Comparisons to healthy controls limit the interpretation of the specificity of findings. For example, while corpus callosum degeneration is regarded as a hallmark of ALS, it is also seen in a range of other neurodegenerative conditions such as HSP, PLS, CBS, etc. [7, 72]. Similarly, imaging papers highlighting anterior cingulate degeneration, reward network and default-mode network dysfunction, amygdala pathology, and hippocampal atrophy in ALS are of interest, but these changes are not specific to ALS either [22]. It seems therefore paramount to include “disease controls” instead of healthy controls to assess the specificity of imaging findings to ALS. Classifier analyses reporting high accuracy in binary classification schemes when distinguishing ALS from healthy controls may have limited clinical relevance, as in “real-life” clinical scenarios it is seldom the question whether someone is healthy or has ALS. Therefore, the inclusion of disease controls and the implementation of multi-class models seem essential to demonstrate the “real-life” diagnostic utility of computational neuroimaging. The cost implications, complex data processing requirements, and poor tolerability of long imaging protocols also need to be acknowledged. As biofluid markers in ALS are increasingly considered informative, cheap, and easily acquired [73, 74], the performance of imaging markers and “wet” biomarkers need to be systematically contrasted to assess their comparative detection, monitoring, and prognostic potential [75]. One of the most challenging facets of ALS imaging is attempting histopathological validation, linking in vivo radiological changes to post-mortem observations [76–78] or co-localising imaging and histological data [79–82]. All of these challenges however are increasingly recognised, and more recent projects carefully address them from the inception.

### New modalities, new techniques, and new MRI platforms

Standard imaging protocols in ALS typically include high-resolution 3D T1-weighted, diffusion MRI and often either T2-wieghted imaging or FLAIR image acquisitions for the visual assessment of microvascular lesion load or comorbid neuroinflammatory changes. Magnetic resonance spectroscopy has long been successfully applied to ALS cohorts [83, 84] and captured motor cortex, thalamic, brainstem, frontotemporal, and hippocampal alterations in the brain [85–89] as well as spinal cord [90] changes. While traditionally implemented as a single-voxel technique, robust multi-voxel MRS papers have also been published in ALS [89, 91]. White matter changes have been traditionally assessed by diffusion MRI implementing various voxelwise and tractography pipelines, but these approaches may be vulnerable to crossing-fibre anatomy. Newer, non-Gaussian diffusion models and other advanced diffusion protocols, such as NODDI, HARDI, and convolution imaging, have brought new insights to our understanding of white matter degeneration in ALS [92–95]. Sodium imaging is another modality which has only been used by a select group of centres and seems to be a useful tool in capturing focal changes [96–98]. Connectomic and graph theory approaches have refined our understanding of network dysfunction in ALS [99]. PET studies have captured both gene-specific [18] and presymptomatic [75] metabolic changes in ALS and combined PET–MR protocols have advanced our understanding of complex pathophysiological processes [75, 100]. Muscle imaging is not routinely performed in ALS, but promising studies have been published [53, 54]. One of the most anticipated technological developments in recent years is the emergence of 7 Tesla MRI platforms. Pilot ALS data has been published from 7 Tesla scanners [82, 101, 102], but the full potential of 7 T imaging is yet to be explored. Another interesting approach is QSM which has been adopted by numerous groups and detected subtle focal changes [103, 104]. Arterial spin labelling is another modality which has only been used by very few centres to date [105, 106]. One of the most important developments in the evolution of scanner technology is that imaging data can be acquired much faster than before which is crucial in ALS, as patients can only tolerate short protocols as the disease advances. Post-mortem imaging has enabled ultrahigh-resolution grey and white matter acquisitions which have refined our understanding of ALS pathology [79, 82]. Co-registration of histopathology and MRI data has improved dramatically and the availability of validated, open-source software and robust free imaging pipelines have also given impetus to ALS research. Access to data repositories such as ADNI has provided an opportunity to test analysis pipelines on non-ALS cohorts. Cloud computing and high-performance multi-core institutional platforms have sped up analyses significantly when a large amount of data has to be pre-processed, spatially registered and segmented. ALS-specific imaging meetings, NISALS, ENCALS, etc., have facilitated informal knowledge exchange and fostered international collaborations. Large multi-centre studies with harmonised protocols have helped to overcome the cohort size limitations of single-centre studies [107–109].

### Machine-learning and cluster analyses

**Fig. 3:**
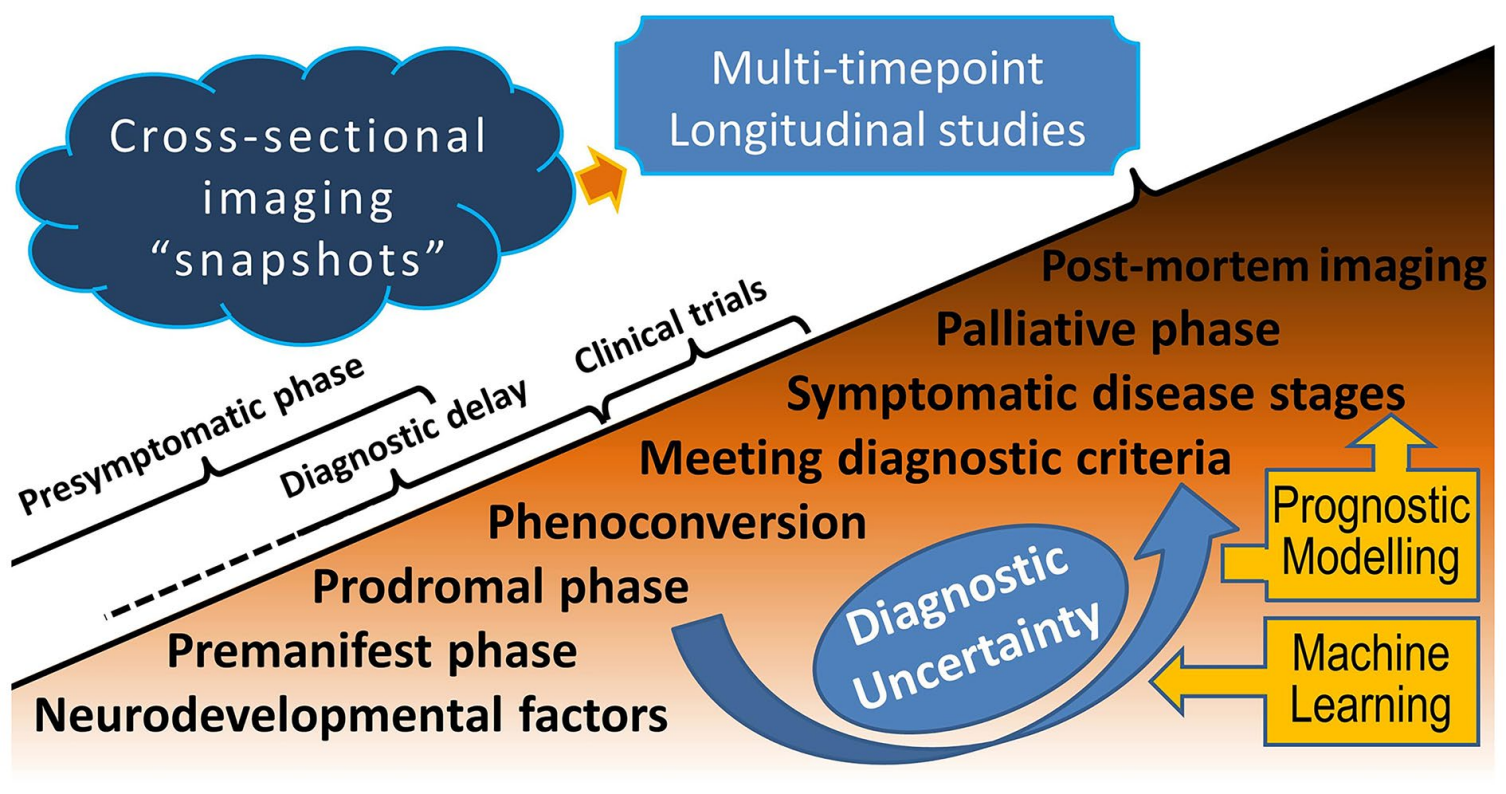
Longitudinal imaging in ALS captures progressive neurodegenerative processes spanning the entire biological course of the disease. Machine-learning frameworks may be developed into viable diagnostic and prognostic applications

Machine-learning (ML) studies have dominated nearly all facets of ALS research in recent years from diagnostic categorisation to prognostication [110–116] (Fig. 3**)**. There is a consensus among ALS neurologists that by the time patients meet diagnostic criteria, considerable degenerative changes have taken place hindering the effectiveness of pharmacological interventions. Diagnostic delay has a considerable literature in ALS and the average interval between symptom onset and formal diagnosis is in the range of 10–14 months [117]. The specific factors contributing to diagnostic delay, the most common misdiagnoses, and the incidence of unnecessary interventions are also well researched [118–120]. The protracted diagnostic journey in ALS has considerable pragmatic ramifications, the chief among which is delayed entry into pharmaceutical trials. One of the purported roles of machine-learning applications in ALS is the confirmation of a suspected diagnosis relatively early based on the recognition of disease-associated biomarker patterns [121] (Fig. 3**)**. After a series of studies reporting good categorisation accuracy in binary classification models [51, 122], recent studies have shown advances in multi-class classification [123, 124]. The majority of recently published studies speculate on the clinical utility of these models and how similar models could be developed into viable diagnostic, monitoring, and prognostic applications. Some have proposed concordance with pathological staging schemes [76, 125], while others focus on prognostication [111, 116]. While the long-term prospects of ML in ALS imaging are clear, the practical challenges are also well recognised. For example, distinguishing early ALS from PLS is notoriously challenging based on brain data alone [124]. ML models risk being overfitted to local data in single-centre studies, and protocol harmonisation is a key barrier to larger multi-site studies. Despite these challenges, a number of promising national [123], single-centre [126], and multi-site studies have been published [116]. A key criterion to appraise the proposed ML initiatives in imaging is whether the model offers additional insights to basic clinical observations, such as, does it predict likely clinical outcomes, survival [110, 116, 127], projected disability, phenoconversion [128], response to therapy, likely genotype for targeted screening, etc. [129]?. Additional clinical roles of ML applications include distinguishing incipient ALS from mimics such as PLS, multifocal motor neuropathy, HSP, etc., and predicting likely disease progression rates [129]. Clearly, recent ML studies in ALS have just started scratching the surface, demonstrating that blinded data sets can be categorised into diagnostic or phenotypic groups relatively accurately, and some prognostic information may be derived, including pathological stage, survival, and phenoconversion [113, 114, 121, 128]. With the emergence of large data sets in ALS, either generated by tertiary referral centres, research consortia, or pharmacological trials, there is also an opportunity to identify small, clinically, or radiologically distinct sub-phenotypes within relatively heterogeneous data samples. A number of studies have performed various cluster analysis approaches to identify subgroups within the clinical spectrum of ALS [130–132]. The classical approach is the stratification of patients by various clinical staging systems and then describing the associated radiological profiles. An alternative, data-driven approach is merging all imaging data irrespective of clinical characteristics, and “let the data talk” by running cluster analyses, identifying “outliers”, and unravelling inherent subgroups with distinctive neuroradiological patterns. Such approaches have revealed unique subgroups with cerebellar disease [130], frontotemporal involvement [131], and frontoparietal dysfunction [132].

### Non-ALS motor neuron diseases

While the neuroimaging literature of MND is dominated by ALS studies, radiological changes in other motor neuron diseases are increasingly well characterised. Large radiological studies have been conducted in spinal and bulbar muscular atrophy (SBMA or Kennedy’s disease) and some cerebral changes have been captured by various research groups [133]. Cerebral and spinal cord changes have been studied in adult SMA, capturing spinal grey matter atrophy as well as cerebral changes [134]. Primary lateral sclerosis (PLS) is one of the best studied non-ALS MNDs, where extensive subcortical, cerebellar, and frontotemporal changes have been described [8, 9, 72, 135]. PLS is also a template condition to study the radiological underpinnings of pseudobulbar affect [41, 136]. The cerebral imaging signature of PLS is very similar to what is observed in ALS, and distinguishing the two based on brain MRI data alone is challenging [124]. Post-poliomyelitis syndrome (PPS) has been traditionally associated with widespread cerebral changes, but recent studies have not only detected limited brain pathology, but also identified increased brainstem, cerebellar, and occipital partial volumes, accompanied by increased fractional anisotropy in the corticospinal tracts [137–139]. Despite the interest in SBMA, PLS, and PPS, the radiological signatures of low-incidence ALS mimics such as Mill’s disease or Hirayama disease are primarily presented as case reports or case series [140–143]. Unfortunately, there is a scarcity of ML studies that would include non-ALS MND phenotypes to test their models’ efficiency in distinguishing these conditions from ALS.

### Supporting clinical observations

**Fig. 4:**
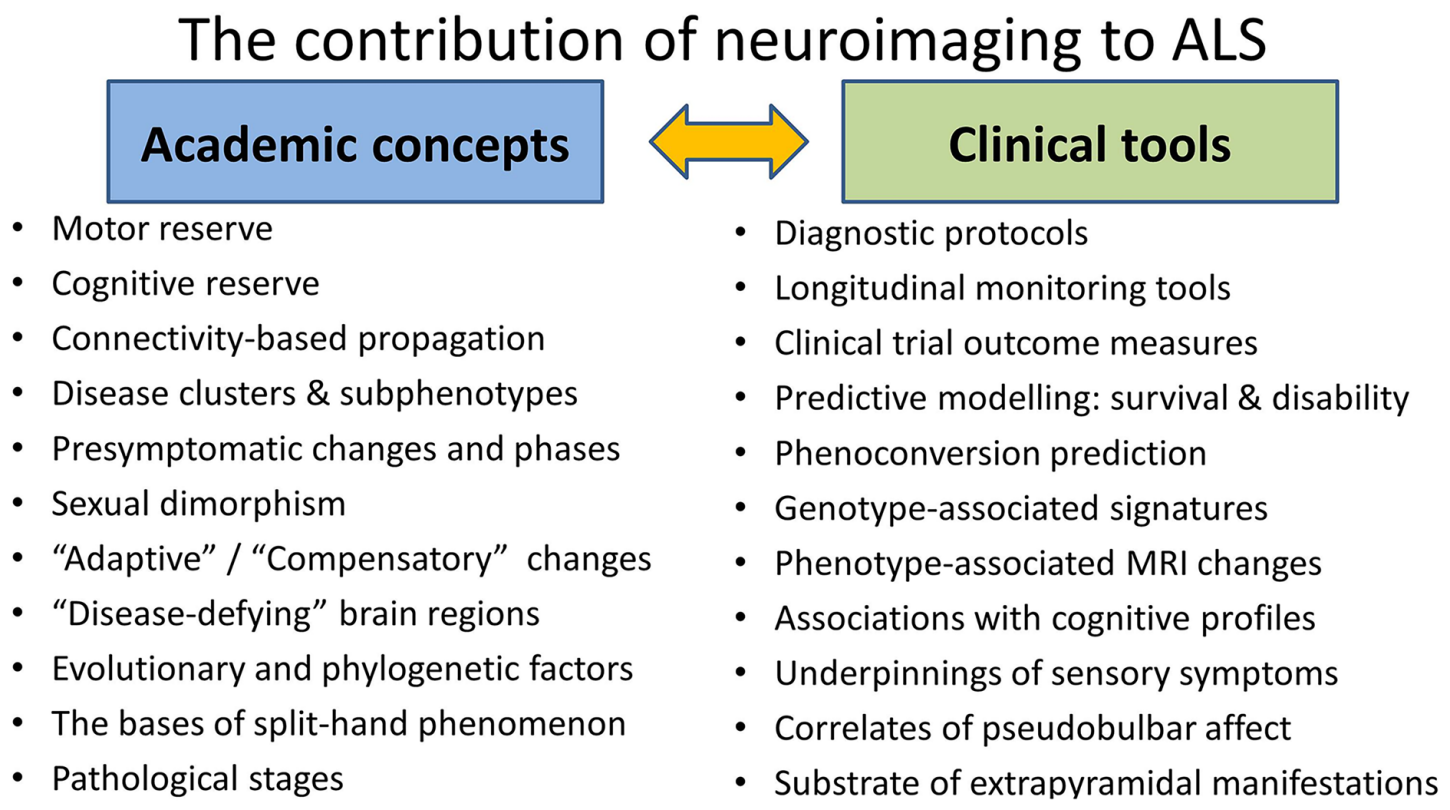
The role of neuroimaging in ALS: testing academic hypotheses and the development of clinical applications

One of the most valuable contributions of neuroimaging is the exploration of the underpinnings of specific clinical manifestations (Fig. 4**)**. Imaging has helped to unravel the pathological bases of behavioural impairment, apathy, extrapyramidal manifestations, respiratory dysfunction, pseudobulbar affect, language impairment, sensory alterations, cerebellar dysfunction, alterations in appetite, weight loss, bulbar symptoms, and spasticity [29, 31, 39, 40, 46, 93, 144, 145]. Neuroimaging has also helped to validate emerging clinical criteria [146, 147], pathological [76, 77, 125, 148, 149], clinical, and cognitive staging systems [14, 43, 150–153], etc. Clinical phenomena such as split-hand and split-leg signs [154] have been explained from an evolutionary perspective [155] and linked to cerebral connectivity patterns [156, 157]. There is also a notion that brain regions that have developed “recently” in evolutionary terms underpinning phylogenetically “novel” skills such as pincer grip, phonation, and executive and language functions are particularly vulnerable to ALS [155, 158, 159] (Fig. 4**)**.

### Other advances from the research field

Specific imaging patterns have been linked to specific phenotypes such as bulbar-onset disease, spinal-onset disease, cognitive phenotypes, and various disease stages. Genotype-specific imaging signatures have been proposed in association with *C9orf72*, *SOD1*, and *ATXN2* [13, 21, 160]. One of the most important research contributions to neuroimaging in ALS is the characterisation of presymptomatic brain and spinal cord changes in asymptomatic mutation carriers [2, 50, 62–64, 161] (Fig. 3**)**. Brain [64] and cord [90] alterations have been described in *SOD1* mutation carriers long before the projected phenoconversion. Spinal cord [50] and brain [62–64] changes have also been detected in association with hexanucleotide repeat expansions in *C9orf72* long before symptom onset. These studies all support the notion that a long presymptomatic phase precedes phenoconversion in ALS [2] and there may also be neurodevelopmental factors at play [161, 162]. More recent studies have shown that neuroimaging data may be helpful in predicting phenoconversion [128], confirming the practical clinical utility of presymptomatic imaging [1]. Academic imaging studies have increasingly evaluated the prevailing biological concepts in ALS such as cognitive reserve [163, 164], motor reserve [165], sexual dimorphism [166, 167], “disease-defying” regions [168], connectivity-based propagation [169], mirror neuron system malfunction [170], spinal interneuron pathology [171, 172], and compensatory and adaptive processes [58]. These studies demonstrate that imaging has an important role in assessing proposed pathophysiological hypotheses in vivo. One of the most important achievements of imaging in ALS is the paradigm shift from focality to connectivity and reconceptualising ALS as a network or circuitrydisease [156, 173]. Imaging studies have traditionally described focal structural changes often with a disproportionate emphasis on the motor cortex, whereas recent studies focus on network-level dysfunction and the progressive structural and functional disconnection between various brain regions. (Fig. 2). Trans-callosal, interhemispheric connectivity has long been described as a core feature of ALS [5], but cerebro-cerebellar, cerebro-thalamic, cerebro-spinal, and cerebro-bulbar disconnection is increasingly recognised as a key facet of ALS pathophysiology [32, 33, 55].

## Future directions, research priorities, and cause for optimism

Despite promising survival and phenoconversion prediction studies [127, 128], MRI-based prognostic modelling is still in its infancy. Future studies should pilot predictive models with regard to the expected age of phenoconversion. Phenotype prediction, i.e. ALS, FTD, or ALS–FTD would also be of clinical relevance in hexanucleotide repeat expansion carriers. A number of insightful spinal studies have been published in ALS, but their real-life utility with regards to monitoring disease progression and distinguishing ALS from PLS, facilitating an earlier diagnosis is yet to be demonstrated [51]. Similarly, a number of elegant studies have now been conducted on 7 Tesla MRI platforms, but its additional value compared to data acquired on 3 Tesla scanners is yet to be demonstrated. While considerable biomarker value has been attributed to MRI-derived metrics [174], the majority of pharmaceutical trials do not consider MRI measures as clinical trial end-points. The development of a reliable pTDP-43 PET tracer would be a landmark achievement, potentially revolutionising our diagnostic and monitoring protocols, but despite promising studies, progress has been relatively slow and a number of challenges remain [175]. Despite the challenges ahead, there is cause for optimism. Data harmonisation efforts have demonstrated the feasibility of large-scale multi-site studies and successful international collaborations have been forged [107–109, 116, 176]. A number of disease-specific consortia now share data and expertise to develop viable clinical tools. Regular international meetings offer knowledge exchange and networking opportunities. Advances in cloud computing, the availability of institutional high-performance computing systems, open-source machine-learning pipelines, and imaging analysis suites have all contributed to effective data processing and meaningful MRI data interpretation in ALS and other MNDs.

## Conclusions

Imaging in ALS and other motor neuron diseases has contributed significantly to our understanding of clinical phenomena and helped to raise awareness of extra-motor manifestations such as frontotemporal, extrapyramidal, and cerebellar dysfunction. The focus of imaging studies has gradually shifted from describing focal brain changes to capturing connectivity alterations and circuitry dysfunction. Dynamic neurodegenerative processes have been detected decades before symptom onset. While academic advances in ALS imaging have not resulted in the development of practical clinical tools, emerging machine-learning studies foreshadow clinically useful diagnostic, prognostic, and monitoring applications.

## References

[1] Benatar M, Wuu J (2012) Presymptomatic studies in ALS: rationale, challenges, and approach. Neurology 79:1732–1739 23071166 10.1212/WNL.0b013e31826e9b1d PMC3468777

[2] Chipika RH, Siah WF, McKenna MC, Li Hi Shing S, Hardiman O, Bede P (2021) The presymptomatic phase of amyotrophic lateral sclerosis: are we merely scratching the surface? J Neurol 268:4607–4629 33130950 10.1007/s00415-020-10289-5

[3] Prudlo J, Bißbort C, Glass A, Grossmann A, Hauenstein K, Benecke R, Teipel SJ (2012) White matter pathology in ALS and lower motor neuron ALS variants: a diffusion tensor imaging study using tract-based spatial statistics. J Neurol 259:1848–1859 22349938 10.1007/s00415-012-6420-y

[4] Schuster C, Elamin M, Hardiman O, Bede P (2016) The segmental diffusivity profile of amyotrophic lateral sclerosis associated white matter degeneration. Eur J Neurol 23:1361–1371 27207250 10.1111/ene.13038

[5] Filippini N, Douaud G, Mackay CE, Knight S, Talbot K, Turner MR (2010) Corpus callosum involvement is a consistent feature of amyotrophic lateral sclerosis. Neurology 75:1645–1652 21041787 10.1212/WNL.0b013e3181fb84d1 PMC2974368

[6] Bede P, Chipika RH, Finegan E, Li Hi Shing S, Doherty MA, Hengeveld JC, Vajda A, Hutchinson S, Donaghy C, McLaughlin RL, Hardiman O (2019) Brainstem pathology in amyotrophic lateral sclerosis and primary lateral sclerosis: a longitudinal neuroimaging study. NeuroImage Clinical 24:102054 31711033 10.1016/j.nicl.2019.102054 PMC6849418

[7] Mulkerrin G, França MC Jr, Lope J, Tan EL, Bede P (2022) Neuroimaging in hereditary spastic paraplegias: from qualitative cues to precision biomarkers. Expert Rev Mol Diagn 22:745–760 36042576 10.1080/14737159.2022.2118048

[8] Finegan E, Li Hi Shing S, Chipika RH, Doherty MA, Hengeveld JC, Vajda A, Donaghy C, Pender N, McLaughlin RL, Hardiman O, Bede P (2019) Widespread subcortical grey matter degeneration in primary lateral sclerosis: a multimodal imaging study with genetic profiling. NeuroImage Clin 24:102089 31795059 10.1016/j.nicl.2019.102089 PMC6978214

[9] Pioro EP, Turner MR, Bede P (2020) Neuroimaging in primary lateral sclerosis. Amyotroph Later Scler Frontotemp Degener 21:18–27 10.1080/21678421.2020.1837176 33602015

[10] Christidi F, Karavasilis E, Velonakis G, Ferentinos P, Rentzos M, Kelekis N, Evdokimidis I, Bede P (2018) The clinical and radiological spectrum of hippocampal pathology in amyotrophic lateral sclerosis. Front Neurol 9:523 30018591 10.3389/fneur.2018.00523 PMC6037820

[11] Spinelli EG, Agosta F, Canu E, Ferraro PM, Riva N, Copetti M, Chiò A, Messina S, Iannaccone S, Calvo A, Silani V, Falini A, Comi G, Filippi M (2014) Cognitive changes and white matter tract damage in the motor neuron disease spectrum. J Neurol 261:S48

[12] Sarro L, Agosta F, Canu E, Riva N, Prelle A, Copetti M, Riccitelli G, Comi G, Filippi M (2011) Cognitive functions and white matter tract damage in amyotrophic lateral sclerosis: a diffusion tensor tractography study. AJNR Am J Neuroradiol 32:1866–1872 22016410 10.3174/ajnr.A2658 PMC7966026

[13] Chipika RH, Christidi F, Finegan E, Li Hi Shing S, McKenna MC, Chang KM, Karavasilis E, Doherty MA, Hengeveld JC, Vajda A, Pender N, Hutchinson S, Donaghy C, McLaughlin RL, Hardiman O, Bede P (2020) Amygdala pathology in amyotrophic lateral sclerosis and primary lateral sclerosis. J Neurol Sci 417:117039 32713609 10.1016/j.jns.2020.117039

[14] Tan HHG, Nitert AD, van Veenhuijzen K, Dukic S, van Zandvoort MJE, Hendrikse J, van Es MA, Veldink JH, Westeneng HJ, van den Berg LH (2025) Neuroimaging correlates of domain-specific cognitive deficits in amyotrophic lateral sclerosis. NeuroImage Clin 45:103749 39947099 10.1016/j.nicl.2025.103749 PMC11869911

[15] Abrahams S, Goldstein LH, Simmons A, Brammer M, Williams SCR, Giampietro V, Leigh PN (2004) Word retrieval in amyotrophic lateral sclerosis: a functional magnetic resonance imaging study. Brain J Neurol 127:1507–1517 10.1093/brain/awh170 15163610

[16] Pettit LD, Bastin ME, Smith C, Bak TH, Gillingwater TH, Abrahams S (2013) Executive deficits, not processing speed relates to abnormalities in distinct prefrontal tracts in amyotrophic lateral sclerosis. Brain J Neurol 136:3290–3304 10.1093/brain/awt243 24056536

[17] McKenna MC, Corcia P, Couratier P, Siah WF, Pradat PF, Bede P (2021) Frontotemporal pathology in motor neuron disease phenotypes: insights from neuroimaging. Front Neurol 12:723450 34484106 10.3389/fneur.2021.723450 PMC8415268

[18] Cistaro A, Pagani M, Montuschi A, Calvo A, Moglia C, Canosa A, Restagno G, Brunetti M, Traynor BJ, Nobili F, Carrara G, Fania P, Lopiano L, Valentini MC, Chio A (2014) The metabolic signature of C9ORF72-related ALS: FDG PET comparison with nonmutated patients. Eur J Nucl Med Mol Imaging. 10.1007/s00259-013-2667-5 24445987 10.1007/s00259-013-2667-5 PMC8957062

[19] Li Hi Shing S, McKenna MC, Siah WF, Chipika RH, Hardiman O, Bede P (2021) The imaging signature of C9orf72 hexanucleotide repeat expansions: implications for clinical trials and therapy development. Brain Imag Behav. 10.1007/s11682-020-00429-w 10.1007/s11682-020-00429-w 33398779

[20] Westeneng HJ, Walhout R, Straathof M, Schmidt R, Hendrikse J, Veldink JH, van den Heuvel MP, van den Berg LH (2016) Widespread structural brain involvement in ALS is not limited to the C9orf72 repeat expansion. J Neurol Neurosurg Psychiatry 87:1354–1360 27756805 10.1136/jnnp-2016-313959 PMC5136726

[21] Chipika RH, Finegan E, Li Hi Shing S, McKenna MC, Christidi F, Chang KM, Doherty MA, Hengeveld JC, Vajda A, Pender N, Hutchinson S, Donaghy C, McLaughlin RL, Hardiman O, Bede P (2020) “Switchboard” malfunction in motor neuron diseases: selective pathology of thalamic nuclei in amyotrophic lateral sclerosis and primary lateral sclerosis. NeuroImage Clin 27:102300 32554322 10.1016/j.nicl.2020.102300 PMC7303672

[22] Christidi F, Kleinerova J, Tan EL, Delaney S, Tacheva A, Hengeveld JC, Doherty MA, McLaughlin RL, Hardiman O, Siah WF, Chang KM, Lope J, Bede P (2024) Limbic network and papez circuit involvement in ALS: imaging and clinical profiles in GGGGCC hexanucleotide carriers in C9orf72 and C9orf72-negative patients. Biology (Basel) 13:504 39056697 10.3390/biology13070504 PMC11273537

[23] Tessitore A, Esposito F, Monsurro MR, Graziano S, Panza D, Russo A, Migliaccio R, Conforti FL, Morrone R, Quattrone A, Di Salle F, Tedeschi G (2006) Subcortical motor plasticity in patients with sporadic ALS: An fMRI study. Brain Res Bull 69:489–494 16647577 10.1016/j.brainresbull.2006.01.013

[24] Trojsi F, Di Nardo F, Caiazzo G, Siciliano M, D’Alvano G, Ferrantino T, Passaniti C, Ricciardi D, Esposito S, Lavorgna L, Russo A, Bonavita S, Cirillo M, Santangelo G, Esposito F, Tedeschi G (2021) Hippocampal connectivity in amyotrophic lateral sclerosis (ALS): more than Papez circuit impairment. Brain Imag Behav 15:2126–2138 10.1007/s11682-020-00408-1 PMC8413176 33095382

[25] Prell T, Grosskreutz J (2013) The involvement of the cerebellum in amyotrophic lateral sclerosis. Amyotrop Later Scler Frontotemp Degener 14:507–515 10.3109/21678421.2013.812661 23889583

[26] Bede P, Chipika RH, Christidi F, Hengeveld JC, Karavasilis E, Argyropoulos GD, Lope J, Li Hi Shing S, Velonakis G, Dupuis L, Doherty MA, Vajda A, McLaughlin RL, Hardiman O (2021) Genotype-associated cerebellar profiles in ALS: focal cerebellar pathology and cerebro-cerebellar connectivity alterations. J Neurol Neurosurg Psychiatry 92:1197–1205 34168085 10.1136/jnnp-2021-326854 PMC8522463

[27] Chipika RH, Mulkerrin G, Pradat PF, Murad A, Ango F, Raoul C, Bede P (2022) Cerebellar pathology in motor neuron disease: neuroplasticity and neurodegeneration. Neural Regen Res 17:2335–2341 35535867 10.4103/1673-5374.336139 PMC9120698

[28] McKenna MC, Chipika RH, Li Hi Shing S, Christidi F, Lope J, Doherty MA, Hengeveld JC, Vajda A, McLaughlin RL, Hardiman O, Hutchinson S, Bede P (2021) Infratentorial pathology in frontotemporal dementia: cerebellar grey and white matter alterations in FTD phenotypes. J Neurol 268:4687–4697 33983551 10.1007/s00415-021-10575-w PMC8563547

[29] Yunusova Y, Plowman EK, Green JR, Barnett C, Bede P (2019) Clinical measures of bulbar dysfunction in ALS. Front Neurol 10:106 30837936 10.3389/fneur.2019.00106 PMC6389633

[30] Van Overwalle F, Manto M, Cattaneo Z, Clausi S, Ferrari C, Gabrieli JDE, Guell X, Heleven E, Lupo M, Ma Q, Michelutti M, Olivito G, Pu M, Rice LC, Schmahmann JD, Siciliano L, Sokolov AA, Stoodley CJ, van Dun K, Vandervert L, Leggio M (2020) Consensus paper: cerebellum and social cognition. The Cerebellum 19:833–868 32632709 10.1007/s12311-020-01155-1 PMC7588399

[31] Chang J, Shaw TB, McCombe PA, Henderson RD, Lucia D, Guo CC, Lv J, Garner K, Bollmann S, Ngo ST, Steyn FJ (2025) Appetite loss in patients with motor neuron disease: impact on weight loss and neural correlates of visual food cues. Brain Commun 7:fcaf111 10.1093/braincomms/fcaf111 PMC11938820 40144301

[32] Tahedl M, Tan EL, Kleinerova J, Delaney S, Hengeveld JC, Doherty MA, McLaughlin RL, Pradat PF, Raoul C, Ango F, Hardiman O, Chang KM, Lope J, Bede P (2024) Progressive cerebrocerebellar uncoupling in sporadic and genetic forms of amyotrophic lateral sclerosis. Neurology 103:e209623 38900989 10.1212/WNL.0000000000209623

[33] Tahedl M, Kleinerova J, Doherty MA, Hengeveld JC, McLaughlin RL, Hardiman O, Tan EL, Bede P (2025) Progressive thalamo-cortical disconnection in amyotrophic lateral sclerosis genotypes: structural degeneration and network dysfunction of thalamus-relayed circuits. Eur J Neurol In Press 10.1111/ene.70146 PMC12064938 40346885

[34] Christidi F, Karavasilis E, Rentzos M, Kelekis N, Evdokimidis I, Bede P (2018) Clinical and radiological markers of extra-motor deficits in amyotrophic lateral sclerosis. Front Neurol 9:1005 30524366 10.3389/fneur.2018.01005 PMC6262087

[35] Feron M, Couillandre A, Mseddi E, Termoz N, Abidi M, Bardinet E, Delgadillo D, Lenglet T, Querin G, Welter ML, Le Forestier N, Salachas F, Bruneteau G, Del Mar AM, Debs R, Lacomblez L, Meininger V, Pelegrini-Issac M, Bede P, Pradat PF, de Marco G (2018) Extrapyramidal deficits in ALS: a combined biomechanical and neuroimaging study. J Neurol 265:2125–2136 29995291 10.1007/s00415-018-8964-y

[36] Abidi M, de Marco G, Grami F, Termoz N, Couillandre A, Querin G, Bede P, Pradat PF (2021) Neural correlates of motor imagery of gait in amyotrophic lateral sclerosis. J Magn Reson Imag 53:223–233 10.1002/jmri.27335 32896088

[37] Bede P, Elamin M, Byrne S, McLaughlin RL, Kenna K, Vajda A, Fagan A, Bradley DG, Hardiman O (2015) Patterns of cerebral and cerebellar white matter degeneration in ALS. J Neurol Neurosurg Psychiatry 86:468–470 25053771 10.1136/jnnp-2014-308172 PMC4392231

[38] Chipika RH, Mulkerrin G, Murad A, Lope J, Hardiman O, Bede P (2022) Alterations in somatosensory, visual and auditory pathways in amyotrophic lateral sclerosis: an under-recognised facet of ALS. J Integr Neurosci 21:88 35633169 10.31083/j.jin2103088

[39] Kleinerova J, Chipika RH, Tan EL, Yunusova Y, Marchand-Pauvert V, Kassubek J, Pradat PF, Bede P (2025) Sensory dysfunction in ALS and other motor neuron diseases: clinical relevance, histopathology, neurophysiology, and insights from neuroimaging. Biomedicines 13 10.3390/biomedicines13030559 PMC11940395 40149536

[40] Trojsi F, Di Nardo F, D’Alvano G, Caiazzo G, Passaniti C, Mangione A, Sharbafshaaer M, Russo A, Silvestro M, Siciliano M, Cirillo M, Tedeschi G, Esposito F (2023) Resting state fMRI analysis of pseudobulbar affect in Amyotrophic lateral sclerosis (ALS): motor dysfunction of emotional expression. Brain Imag Behav 17:77–89 10.1007/s11682-022-00744-4 PMC9922228 36370302

[41] Tahedl M, Tan EL, Siah WF, Hengeveld JC, Doherty MA, McLaughlin RL, Hardiman O, Finegan E, Bede P (2023) Radiological correlates of pseudobulbar affect: corticobulbar and cerebellar components in primary lateral sclerosis. J Neurol Sci 451:120726 37421883 10.1016/j.jns.2023.120726

[42] Aho-Ozhan HE, Keller J, Heimrath J, Uttner I, Kassubek J, Birbaumer N, Ludolph AC, Lule D (2016) Perception of emotional facial expressions in Amyotrophic lateral sclerosis (ALS) at behavioural and brain metabolic level. PLoS One 11:e0164655 27741285 10.1371/journal.pone.0164655 PMC5065224

[43] Burke T, Elamin M, Bede P, Pinto-Grau M, Lonergan K, Hardiman O, Pender N (2016) Discordant performance on the ‘Reading the Mind in the Eyes’ Test, based on disease onset in amyotrophic lateral sclerosis. Amyotrop Later Scler Frontotemp Degener 17:467–472 10.1080/21678421.2016.1177088 27152765

[44] Radakovic R, Stephenson L, Colville S, Swingler R, Chandran S, Abrahams S (2016) Multidimensional apathy in ALS: validation of the dimensional apathy scale. J Neurol Neurosurg Psychiatry 87:663–669 26203157 10.1136/jnnp-2015-310772

[45] Gorges M, Vercruysse P, Muller HP, Huppertz HJ, Rosenbohm A, Nagel G, Weydt P, Petersen A, Ludolph AC, Kassubek J, Dupuis L (2017) Hypothalamic atrophy is related to body mass index and age at onset in amyotrophic lateral sclerosis. J Neurol Neurosurg Psychiatry 88:1033–1041 28596251 10.1136/jnnp-2017-315795

[46] Chang J, Shaw TB, Holdom CJ, McCombe PA, Henderson RD, Fripp J, Barth M, Guo CC, Ngo ST, Steyn FJ (2023) Lower hypothalamic volume with lower body mass index is associated with shorter survival in patients with amyotrophic lateral sclerosis. Eur J Neurol 30:57–68 36214080 10.1111/ene.15589 PMC10099625

[47] Michielsen A, van Veenhuijzen K, Janse van Mantgem MR, van Es MA, Veldink JH, van Eijk RPA, van den Berg LH, Westeneng HJ (2024) Association between hypothalamic volume and metabolism, cognition, and behavior in patients with amyotrophic lateral sclerosis. Neurology 103:e209603 38875517 10.1212/WNL.0000000000209603 PMC11244736

[48] El Mendili MM, Querin G, Bede P, Pradat PF (2019) Spinal cord imaging in amyotrophic lateral sclerosis: historical concepts-novel techniques. Front Neurol 10:350 31031688 10.3389/fneur.2019.00350 PMC6474186

[49] Bede P, Bokde AL, Byrne S, Elamin M, Fagan AJ, Hardiman O (2012) Spinal cord markers in ALS: diagnostic and biomarker considerations. Amyotrop Later Scler 13:407–415 10.3109/17482968.2011.649760 22329869

[50] Querin G, Bede P, El Mendili MM, Li M, Pelegrini-Issac M, Rinaldi D, Catala M, Saracino D, Salachas F, Camuzat A, Marchand-Pauvert V, Cohen-Adad J, Colliot O, Le Ber I, Pradat PF (2019) Presymptomatic spinal cord pathology in c9orf72 mutation carriers: a longitudinal neuroimaging study. Ann Neurol 86:158–167 31177556 10.1002/ana.25520

[51] Querin G, El Mendili MM, Bede P, Delphine S, Lenglet T, Marchand-Pauvert V, Pradat PF (2018) Multimodal spinal cord MRI offers accurate diagnostic classification in ALS. J Neurol Neurosurg Psychiatry 89:1220–1221 29353238 10.1136/jnnp-2017-317214

[52] Cohen-Adad J, El Mendili MM, Morizot-Koutlidis R, Lehericy S, Meininger V, Blancho S, Rossignol S, Benali H, Pradat PF (2013) Involvement of spinal sensory pathway in ALS and specificity of cord atrophy to lower motor neuron degeneration. Amyotroph Later Scler Frontotemp Degener 14:30–38 10.3109/17482968.2012.701308 22881412

[53] Jenkins TM, Alix JJP, David C, Pearson E, Rao DG, Hoggard N, O’Brien E, Baster K, Bradburn M, Bigley J, McDermott CJ, Wilkinson ID, Shaw PJ (2017) Imaging muscle as a potential biomarker of denervation in motor neuron disease. J Neurol Neurosurg Psychiatry 10.1136/jnnp-2017-316744 PMC5869448 29089397

[54] Jenkins TM, Burness C, Connolly DJ, Rao DG, Hoggard N, Mawson S, McDermott CJ, Wilkinson ID, Shaw PJ (2013) A prospective pilot study measuring muscle volumetric change in amyotrophic lateral sclerosis. Amyotroph Later Scler Frontotemp Degener 14:414–423 10.3109/21678421.2013.795597 23705876

[55] Tahedl M, Tan EL, Chipika RH, Hengeveld JC, Vajda A, Doherty MA, McLaughlin RL, Siah WF, Hardiman O, Bede P (2023) Brainstem-cortex disconnection in amyotrophic lateral sclerosis: bulbar impairment, genotype associations, asymptomatic changes and biomarker opportunities. J Neurol 270:3511–3526 37022479 10.1007/s00415-023-11682-6 PMC10267265

[56] Pinto S, Alves P, Pimentel B, Swash M, de Carvalho M (2016) Ultrasound for assessment of diaphragm in ALS. Clin Neurophysiol 127:892–897 25971723 10.1016/j.clinph.2015.03.024

[57] Menke RA, Proudfoot M, Wuu J, Andersen PM, Talbot K, Benatar M, Turner MR (2016) Increased functional connectivity common to symptomatic amyotrophic lateral sclerosis and those at genetic risk. J Neurol Neurosurg Psychiatry 87:580–588 26733601 10.1136/jnnp-2015-311945 PMC4893149

[58] Abidi M, de Marco G, Couillandre A, Feron M, Mseddi E, Termoz N, Querin G, Pradat PF, Bede P (2020) Adaptive functional reorganization in amyotrophic lateral sclerosis: coexisting degenerative and compensatory changes. Eur J Neurol 27:121–128 31310452 10.1111/ene.14042

[59] Cistaro A, Valentini MC, Chio A, Nobili F, Calvo A, Moglia C, Montuschi A, Morbelli S, Salmaso D, Fania P, Carrara G, Pagani M (2012) Brain hypermetabolism in amyotrophic lateral sclerosis: a FDG PET study in ALS of spinal and bulbar onset. Eur J Nucl Med Mol Imag 39:251–259 10.1007/s00259-011-1979-6 22089661

[60] Kleinerova J, Tahedl M, McKenna MC, Garcia-Gallardo A, Hutchinson S, Hardiman O, Raoul C, Ango F, Schneider B, Pradat PF, Tan EL, Bede P (2025) Cerebellar dysfunction in frontotemporal dementia: intra-cerebellar pathology and cerebellar network degeneration. J Neurol 272:289 40131525 10.1007/s00415-025-13046-8 PMC11937067

[61] Verstraete E, Turner MR, Grosskreutz J, Filippi M, Benatar M (2015) Mind the gap: the mismatch between clinical and imaging metrics in ALS. Amyotroph Later Scler Frontotemp Degener 16:524–529 10.3109/21678421.2015.1051989 26402254

[62] Wen J, Zhang H, Alexander DC, Durrleman S, Routier A, Rinaldi D, Houot M, Couratier P, Hannequin D, Pasquier F, Zhang J, Colliot O, Le Ber I, Bertrand A (2019) Neurite density is reduced in the presymptomatic phase of C9orf72 disease. J Neurol Neurosurg Psychiatry 90:387–394 30355607 10.1136/jnnp-2018-318994

[63] Bertrand A, Wen J, Rinaldi D, Houot M, Sayah S, Camuzat A, Fournier C, Fontanella S, Routier A, Couratier P, Pasquier F, Habert MO, Hannequin D, Martinaud O, Caroppo P, Levy R, Dubois B, Brice A, Durrleman S, Colliot O, Le Ber I (2018) Early cognitive, structural, and microstructural changes in presymptomatic C9orf72 carriers younger than 40 years. JAMA Neurol 75:236–245 29197216 10.1001/jamaneurol.2017.4266 PMC5838615

[64] Bede P, Lulé D, Müller HP, Tan EL, Dorst J, Ludolph AC, Kassubek J (2023) Presymptomatic grey matter alterations in ALS kindreds: a computational neuroimaging study of asymptomatic C9orf72 and SOD1 mutation carriers. J Neurol. 10.1007/s00415-023-11764-5 37178170 10.1007/s00415-023-11764-5 PMC10421803

[65] Metzger M, Dukic S, McMackin R, Giglia E, Mitchell M, Bista S, Costello E, Peelo C, Tadjine Y, Sirenko V, Plaitano S, Coffey A, McManus L, Farnell Sharp A, Mehra P, Heverin M, Bede P, Muthuraman M, Pender N, Hardiman O, Nasseroleslami B (2024) Functional network dynamics revealed by EEG microstates reflect cognitive decline in amyotrophic lateral sclerosis. Hum Brain Mapp 45:e26536 38087950 10.1002/hbm.26536 PMC10789208

[66] Iyer PM, Mohr K, Broderick M, Gavin B, Burke T, Bede P, Pinto-Grau M, Pender NP, McLaughlin R, Vajda A, Heverin M, Lalor EC, Hardiman O, Nasseroleslami B (2017) Mismatch negativity as an indicator of cognitive sub-domain dysfunction in amyotrophic lateral sclerosis. Front Neurol 8:395 28861032 10.3389/fneur.2017.00395 PMC5559463

[67] Nasseroleslami B, Dukic S, Broderick M, Mohr K, Schuster C, Gavin B, McLaughlin R, Heverin M, Vajda A, Iyer PM, Pender N, Bede P, Lalor EC, Hardiman O (2019) Characteristic increases in EEG connectivity correlate with changes of structural MRI in amyotrophic lateral sclerosis. Cereb Cortex 29:27–41 29136131 10.1093/cercor/bhx301

[68] Proudfoot M, Bede P, Turner MR (2018) Imaging cerebral activity in amyotrophic lateral sclerosis. Front Neurol 9:1148 30671016 10.3389/fneur.2018.01148 PMC6332509

[69] Proudfoot M, Rohenkohl G, Quinn A, Colclough GL, Wuu J, Talbot K, Woolrich MW, Benatar M, Nobre AC, Turner MR (2017) Altered cortical beta-band oscillations reflect motor system degeneration in amyotrophic lateral sclerosis. Hum Brain Mapp 38:237–254 27623516 10.1002/hbm.23357 PMC5215611

[70] Schuster C, Elamin M, Hardiman O, Bede P (2015) Presymptomatic and longitudinal neuroimaging in neurodegeneration–from snapshots to motion picture: a systematic review. J Neurol Neurosurg Psychiatry 86:1089–1096 25632156 10.1136/jnnp-2014-309888

[71] Tahedl M, Li Hi Shing S, Finegan E, Chipika RH, Lope J, Hardiman O, Bede P (2021) Propagation patterns in motor neuron diseases: Individual and phenotype-associated disease-burden trajectories across the UMN-LMN spectrum of MNDs. Neurobiol Aging 109:78–87 34656922 10.1016/j.neurobiolaging.2021.04.031

[72] Finegan E, Shing SLH, Chipika RH, Chang KM, McKenna MC, Doherty MA, Hengeveld JC, Vajda A, Pender N, Donaghy C, Hutchinson S, McLaughlin RL, Hardiman O, Bede P (2021) Extra-motor cerebral changes and manifestations in primary lateral sclerosis. Brain Imaging Behav 15:2283–2296 33409820 10.1007/s11682-020-00421-4

[73] Blasco H, Patin F, Descat A, Garcon G, Corcia P, Gele P, Lenglet T, Bede P, Meininger V, Devos D, Gossens JF, Pradat PF (2018) A pharmaco-metabolomics approach in a clinical trial of ALS: Identification of predictive markers of progression. PLoS One 13:e0198116 29870556 10.1371/journal.pone.0198116 PMC5988280

[74] Devos D, Moreau C, Kyheng M, Garcon G, Rolland AS, Blasco H, Gele P, Timothee Lenglet T, Veyrat-Durebex C, Corcia P, Dutheil M, Bede P, Jeromin A, Oeckl P, Otto M, Meninger V, Danel-Brunaud V, Devedjian JC, Duce JA, Pradat PF (2019) A ferroptosis-based panel of prognostic biomarkers for amyotrophic lateral sclerosis. Sci Rep 9:2918 30814647 10.1038/s41598-019-39739-5 PMC6393674

[75] De Vocht J, Blommaert J, Devrome M, Radwan A, Van Weehaeghe D, De Schaepdryver M, Ceccarini J, Rezaei A, Schramm G, van Aalst J, Chiò A, Pagani M, Stam D, Van Esch H, Lamaire N, Verhaegen M, Mertens N, Poesen K, van den Berg LH, van Es MA, Vandenberghe R, Vandenbulcke M, Van den Stock J, Koole M, Dupont P, Van Laere K, Van Damme P (2020) Use of multimodal imaging and clinical biomarkers in presymptomatic carriers of C9orf72 repeat expansion. JAMA Neurol 77:1008–1017 32421156 10.1001/jamaneurol.2020.1087 PMC7417970

[76] Behler A, Müller HP, Del Tredici K, Braak H, Ludolph AC, Lulé D, Kassubek J (2022) Multimodal in vivo staging in amyotrophic lateral sclerosis using artificial intelligence. Annals Clin Trans Neurol 9:1069–1079 10.1002/acn3.51601 PMC9268886 35684940

[77] Geser F, Prvulovic D, O’Dwyer L, Hardiman O, Bede P, Bokde AL, Trojanowski JQ, Hampel H (2011) On the development of markers for pathological TDP-43 in amyotrophic lateral sclerosis with and without dementia. Prog Neurobiol 95:649–662 21911035 10.1016/j.pneurobio.2011.08.011 PMC3230745

[78] Bede P (2019) The histological correlates of imaging metrics: postmortem validation of in vivo findings. Amyotroph Later Scler Frontotemp Degener 20:457–460 10.1080/21678421.2019.1639195 31293187

[79] Pallebage-Gamarallage M, Foxley S, Menke RAL, Huszar IN, Jenkinson M, Tendler BC, Wang C, Jbabdi S, Turner MR, Miller KL, Ansorge O (2018) Dissecting the pathobiology of altered MRI signal in amyotrophic lateral sclerosis: a post mortem whole brain sampling strategy for the integration of ultra-high-field MRI and quantitative neuropathology. BMC Neurosci 19:11 29529995 10.1186/s12868-018-0416-1 PMC5848544

[80] Kor DZL, Jbabdi S, Huszar IN, Mollink J, Tendler BC, Foxley S, Wang C, Scott C, Smart A, Ansorge O, Pallebage-Gamarallage M, Miller KL, Howard AFD (2022) An automated pipeline for extracting histological stain area fraction for voxelwise quantitative MRI-histology comparisons. Neuroimage 264:119726 36368503 10.1016/j.neuroimage.2022.119726 PMC10933753

[81] Wang C, Foxley S, Ansorge O, Bangerter-Christensen S, Chiew M, Leonte A, Menke RA, Mollink J, Pallebage-Gamarallage M, Turner MR, Miller KL, Tendler BC (2020) Methods for quantitative susceptibility and R2* mapping in whole post-mortem brains at 7T applied to amyotrophic lateral sclerosis. Neuroimage 222:117216 32745677 10.1016/j.neuroimage.2020.117216 PMC7775972

[82] Cardenas AM, Sarlls JE, Kwan JY, Bageac D, Gala ZS, Danielian LE, Ray-Chaudhury A, Wang HW, Miller KL, Foxley S, Jbabdi S, Welsh RC, Floeter MK (2017) Pathology of callosal damage in ALS: An ex-vivo, 7 T diffusion tensor MRI study. NeuroImage Clinical 15:200–208 28529876 10.1016/j.nicl.2017.04.024 PMC5429246

[83] Pioro EP (1997) MR spectroscopy in amyotrophic lateral sclerosis/motor neuron disease. J Neurol Sci 152(Suppl 1):S49–S53 9419054 10.1016/s0022-510x(97)00244-x

[84] Kalra S, Cashman NR, Genge A, Arnold DL (1998) Recovery of N-acetylaspartate in corticomotor neurons of patients with ALS after riluzole therapy. NeuroReport 9:1757–1761 9665596 10.1097/00001756-199806010-00016

[85] Christidi F, Karavasilis E, Argyropoulos GD, Velonakis G, Zouvelou V, Murad A, Evdokimidis I, Rentzos M, Seimenis I, Bede P (2022) Neurometabolic alterations in motor neuron disease: insights from magnetic resonance spectroscopy. J Integr Neurosci 21:87 35633168 10.31083/j.jin2103087

[86] Christidi F, Argyropoulos GD, Karavasilis E, Velonakis G, Zouvelou V, Kourtesis P, Pantoleon V, Tan EL, Daponte A, Aristeidou S, Xirou S, Ferentinos P, Evdokimidis I, Rentzos M, Seimenis I, Bede P (2023) Hippocampal metabolic alterations in amyotrophic lateral sclerosis: a magnetic resonance spectroscopy study. Life (Basel, Switzerland) 13 10.3390/life13020571 PMC9965919 36836928

[87] Kalra S, Hanstock CC, Martin WR, Allen PS, Johnston WS (2006) Detection of cerebral degeneration in amyotrophic lateral sclerosis using high-field magnetic resonance spectroscopy. Arch Neurol 63:1144–1148 16908742 10.1001/archneur.63.8.1144

[88] Sharma KR, Saigal G, Maudsley AA, Govind V (2011) 1H MRS of basal ganglia and thalamus in amyotrophic lateral sclerosis. NMR Biomed 24:1270–1276 21404355 10.1002/nbm.1687 PMC3210902

[89] Govind V, Sharma KR, Maudsley AA, Arheart KL, Saigal G, Sheriff S (2012) Comprehensive evaluation of corticospinal tract metabolites in amyotrophic lateral sclerosis using whole-brain 1H MR spectroscopy. PLoS One 7:e35607 22539984 10.1371/journal.pone.0035607 PMC3335096

[90] Carew JD, Nair G, Andersen PM, Wuu J, Gronka S, Hu X, Benatar M (2011) Presymptomatic spinal cord neurometabolic findings in SOD1-positive people at risk for familial ALS. Neurology 77:1370–1375 21940617 10.1212/WNL.0b013e318231526a PMC3182757

[91] Stagg CJ, Knight S, Talbot K, Jenkinson M, Maudsley AA, Turner MR (2013) Whole-brain magnetic resonance spectroscopic imaging measures are related to disability in ALS. Neurology 80:610–615 23325907 10.1212/WNL.0b013e318281ccec PMC3590062

[92] Barritt AW, Gabel MC, Cercignani M, Leigh PN (2018) Emerging magnetic resonance imaging techniques and analysis methods in amyotrophic lateral sclerosis. Front Neurol 9:1065 30564192 10.3389/fneur.2018.01065 PMC6288229

[93] Tahedl M, Tan EL, Chipika RH, Lope J, Hengeveld JC, Doherty MA, McLaughlin RL, Hardiman O, Hutchinson S, McKenna MC, Bede P (2023) The involvement of language-associated networks, tracts, and cortical regions in frontotemporal dementia and amyotrophic lateral sclerosis: Structural and functional alterations. Brain Behav. 10.1002/brb3.3250 37694825 10.1002/brb3.3250 PMC10636407

[94] Tahedl M, Murad A, Lope J, Hardiman O, Bede P (2021) Evaluation and categorisation of individual patients based on white matter profiles: Single-patient diffusion data interpretation in neurodegeneration. J Neurol Sci 428:117584 34315000 10.1016/j.jns.2021.117584

[95] Trojsi F, Caiazzo G, Di Nardo F, Fratello M, Santangelo G, Siciliano M, Femiano C, Russo A, Monsurro MR, Cirillo M, Tedeschi G, Esposito F (2017) High angular resolution diffusion imaging abnormalities in the early stages of amyotrophic lateral sclerosis. J Neurol Sci 380:215–222 28870572 10.1016/j.jns.2017.07.039

[96] Grapperon AM, Ridley B, Verschueren A, Maarouf A, Confort-Gouny S, Fortanier E, Schad L, Guye M, Ranjeva JP, Attarian S, Zaaraoui W (2019) Quantitative brain sodium MRI depicts corticospinal impairment in amyotrophic lateral sclerosis. Radiology 292:422–428 31184559 10.1148/radiol.2019182276

[97] El Mendili MM, Grapperon AM, Dintrich R, Stellmann JP, Ranjeva JP, Guye M, Verschueren A, Attarian S, Zaaraoui W (2022) Alterations of microstructure and sodium homeostasis in fast amyotrophic lateral sclerosis progressors: a brain DTI and sodium MRI study. AJNR Am J Neuroradiol 43:984–990 35772800 10.3174/ajnr.A7559 PMC9262065

[98] Grapperon AM, El Mendili MM, Maarouf A, Ranjeva JP, Guye M, Verschueren A, Attarian S, Zaaraoui W (2025) In vivo mapping of sodium homeostasis disturbances in individual ALS patients: A brain 23Na MRI study. PLoS One 20:e0316916 39841674 10.1371/journal.pone.0316916 PMC11753670

[99] Rajagopalan V, Pioro EP (2022) Graph theory network analysis provides brain MRI evidence of a partial continuum of neurodegeneration in patients with UMN-predominant ALS and ALS-FTD. NeuroImage Clin 35:103037 35597032 10.1016/j.nicl.2022.103037 PMC9123271

[100] Juengling FD, Wuest F, Kalra S, Agosta F, Schirrmacher R, Thiel A, Thaiss W, Müller HP, Kassubek J (2022) Simultaneous PET/MRI: The future gold standard for characterizing motor neuron disease-A clinico-radiological and neuroscientific perspective. Front Neurol 13:890425 36061999 10.3389/fneur.2022.890425 PMC9428135

[101] Verstraete E, Biessels GJ, van Den Heuvel MP, Visser F, Luijten PR, van Den Berg LH (2010) No evidence of microbleeds in ALS patients at 7 Tesla MRI. Amyotrop Later Scler 11:555–557 10.3109/17482968.2010.513053 20812888

[102] Barry RL, Babu S, Anteraper SA, Triantafyllou C, Keil B, Rowe OE, Rangaprakash D, Paganoni S, Lawson R, Dheel C, Cernasov PM, Rosen BR, Ratai EM, Atassi N (2021) Ultra-high field (7T) functional magnetic resonance imaging in amyotrophic lateral sclerosis: a pilot study. NeuroImage Clin 30:102648 33872993 10.1016/j.nicl.2021.102648 PMC8060594

[103] Acosta-Cabronero J, Machts J, Schreiber S, Abdulla S, Kollewe K, Petri S, Spotorno N, Kaufmann J, Heinze HJ, Dengler R, Vielhaber S, Nestor PJ (2018) Quantitative susceptibility MRI to detect brain iron in amyotrophic lateral sclerosis. Radiology 289:195–203 30040038 10.1148/radiol.2018180112 PMC6166868

[104] Costagli M, Donatelli G, Biagi L, Caldarazzo Ienco E, Siciliano G, Tosetti M, Cosottini M (2016) Magnetic susceptibility in the deep layers of the primary motor cortex in Amyotrophic Lateral Sclerosis. NeuroImage Clin 12:965–969 27995062 10.1016/j.nicl.2016.04.011 PMC5153607

[105] Canna A, Trojsi F, Di Nardo F, Caiazzo G, Tedeschi G, Cirillo M, Esposito F (2021) Combining structural and metabolic markers in a quantitative MRI study of motor neuron diseases. Annals Clin Trans Neurol 8:1774–1785 10.1002/acn3.51418 PMC8419394 34342169

[106] Wang Y, Shen D, Hou B, Sun X, Yang X, Gao J, Liu M, Feng F, Cui L (2022) Brain structural and perfusion changes in amyotrophic lateral sclerosis-frontotemporal dementia patients with cognitive and motor onset: a preliminary study. Brain Imaging Behav 16:2164–2174 35838935 10.1007/s11682-022-00686-x

[107] Bharti K, Khan M, Beaulieu C, Graham SJ, Briemberg H, Frayne R, Genge A, Korngut L, Zinman L, Kalra S (2020) Involvement of the dentate nucleus in the pathophysiology of amyotrophic lateral sclerosis: a multi-center and multi-modal neuroimaging study. NeuroImage Clin 28:102385 32871387 10.1016/j.nicl.2020.102385 PMC7476068

[108] Bharti K, Graham S, Benatar M, Briemberg H, Chenji S, Dupré N, Dionne A, Frayne R, Genge A, Korngut L, Luk C, Zinman L, Kalra S (2022) Functional alterations in large-scale resting-state networks of amyotrophic lateral sclerosis: a multi-site study across Canada and the United States. PLoS One 17:e0269154 35709100 10.1371/journal.pone.0269154 PMC9202847

[109] Dey A, Luk CC, Ishaque A, Ta D, Srivastava O, Krebs D, Seres P, Hanstock C, Beaulieu C, Korngut L, Frayne R, Zinman L, Graham S, Genge A, Briemberg H, Kalra S (2022) Motor cortex functional connectivity is associated with underlying neurochemistry in ALS. J Neurol Neurosurg Psychiatry. 94(3):193–200 36379713 10.1136/jnnp-2022-329993 PMC9985743

[110] Westeneng HJ, Debray TPA, Visser AE, van Eijk RPA, Rooney JPK, Calvo A, Martin S, McDermott CJ, Thompson AG, Pinto S, Kobeleva X, Rosenbohm A, Stubendorff B, Sommer H, Middelkoop BM, Dekker AM, van Vugt J, van Rheenen W, Vajda A, Heverin M, Kazoka M, Hollinger H, Gromicho M, Körner S, Ringer TM, Rödiger A, Gunkel A, Shaw CE, Bredenoord AL, van Es MA, Corcia P, Couratier P, Weber M, Grosskreutz J, Ludolph AC, Petri S, de Carvalho M, Van Damme P, Talbot K, Turner MR, Shaw PJ, Al-Chalabi A, Chiò A, Hardiman O, Moons KGM, Veldink JH, van den Berg LH (2018) Prognosis for patients with amyotrophic lateral sclerosis: development and validation of a personalised prediction model. Lancet Neurol 17:423–433 29598923 10.1016/S1474-4422(18)30089-9

[111] van der Burgh HK, Schmidt R, Westeneng HJ, de Reus MA, van den Berg LH, van den Heuvel MP (2017) Deep learning predictions of survival based on MRI in amyotrophic lateral sclerosis. NeuroImage Clin 13:361–369 28070484 10.1016/j.nicl.2016.10.008 PMC5219634

[112] Schuster C, Hardiman O, Bede P (2016) Development of an automated mri-based diagnostic protocol for amyotrophic lateral sclerosis using disease-specific pathognomonic features: a quantitative disease-state classification study. PLoS One 11:e0167331 27907080 10.1371/journal.pone.0167331 PMC5132189

[113] Grollemund V, Chat GL, Secchi-Buhour MS, Delbot F, Pradat-Peyre JF, Bede P, Pradat PF (2020) Development and validation of a 1-year survival prognosis estimation model for amyotrophic lateral sclerosis using manifold learning algorithm UMAP. Sci Rep 10:13378 32770027 10.1038/s41598-020-70125-8 PMC7414917

[114] Grollemund V, Le Chat G, Secchi-Buhour MS, Delbot F, Pradat-Peyre JF, Bede P, Pradat PF (2021) Manifold learning for amyotrophic lateral sclerosis functional loss assessment: development and validation of a prognosis model. J Neurol 268:825–850 32886252 10.1007/s00415-020-10181-2

[115] Behler A, Müller HP, Ludolph AC, Kassubek J (2023) Diffusion tensor imaging in amyotrophic lateral sclerosis: machine learning for biomarker development. Int J Mol Sci 24(3):1911 10.3390/ijms24031911 PMC9915541 36768231

[116] Lajoie I, Kalra S, Dadar M (2025) Regional cerebral atrophy contributes to personalized survival prediction in amyotrophic lateral sclerosis: a multicentre, machine learning, deformation-based morphometry study. Annals Neurol 10.1002/ana.27196 PMC12082021 39985309

[117] Cellura E, Spataro R, Taiello AC, La Bella V (2012) Factors affecting the diagnostic delay in amyotrophic lateral sclerosis. Clin Neurol Neurosurg 114:550–554 22169158 10.1016/j.clineuro.2011.11.026

[118] Zoccolella S, Beghi E, Palagano G, Fraddosio A, Samarelli V, Lamberti P, Lepore V, Serlenga L, Logroscino G (2006) Predictors of delay in the diagnosis and clinical trial entry of amyotrophic lateral sclerosis patients: a population-based study. J Neurol Sci 250:45–49 16920152 10.1016/j.jns.2006.06.027

[119] Chio A, Logroscino G, Hardiman O, Swingler R, Mitchell D, Beghi E, Traynor BG (2009) Prognostic factors in ALS: a critical review. Amyotroph Later Scler 10:310–323 10.3109/17482960802566824 PMC3515205 19922118

[120] Kraemer M, Buerger M, Berlit P (2010) Diagnostic problems and delay of diagnosis in amyotrophic lateral sclerosis. Clin Neurol Neurosurg 112:103–105 19931253 10.1016/j.clineuro.2009.10.014

[121] Grollemund V, Pradat PF, Querin G, Delbot F, Le Chat G, Pradat-Peyre JF, Bede P (2019) Machine learning in amyotrophic lateral sclerosis: achievements, pitfalls, and future directions. Front Neurosci 13:135 30872992 10.3389/fnins.2019.00135 PMC6403867

[122] Bede P, Iyer PM, Finegan E, Omer T, Hardiman O (2017) Virtual brain biopsies in amyotrophic lateral sclerosis: Diagnostic classification based on in vivo pathological patterns. NeuroImage Clinical 15:653–658 28664036 10.1016/j.nicl.2017.06.010 PMC5479963

[123] Bede P, Murad A, Hardiman O (2021) Pathological neural networks and artificial neural networks in ALS: diagnostic classification based on pathognomonic neuroimaging features. J Neurol. 10.1007/s00415-021-10801-5 34585269 10.1007/s00415-021-10801-5 PMC9021106

[124] Bede P, Murad A, Lope J, Li Hi Shing S, Finegan E, Chipika RH, Hardiman O, Chang KM (2021) Phenotypic categorisation of individual subjects with motor neuron disease based on radiological disease burden patterns: a machine-learning approach. J Neurol Sci 432:120079 34875472 10.1016/j.jns.2021.120079

[125] Behler A, Müller HP, Ludolph AC, Lulé D, Kassubek J (2022) A multivariate Bayesian classification algorithm for cerebral stage prediction by diffusion tensor imaging in amyotrophic lateral sclerosis. NeuroImage Clin 35:103094 35772192 10.1016/j.nicl.2022.103094 PMC9253469

[126] Welsh RC, Jelsone-Swain LM, Foerster BR (2013) The utility of independent component analysis and machine learning in the identification of the amyotrophic lateral sclerosis diseased brain. Front Hum Neurosci 7:251 23772210 10.3389/fnhum.2013.00251 PMC3677153

[127] Schuster C, Hardiman O, Bede P (2017) Survival prediction in Amyotrophic lateral sclerosis based on MRI measures and clinical characteristics. BMC Neurol 17:73 28412941 10.1186/s12883-017-0854-x PMC5393027

[128] van Veenhuijzen K, Tan HHG, Nitert AD, van Es MA, Veldink JH, van den Berg LH, Westeneng HJ (2025) Longitudinal magnetic resonance imaging in asymptomatic C9orf72 mutation carriers distinguishes phenoconverters to amyotrophic lateral sclerosis or amyotrophic lateral sclerosis with frontotemporal dementia. Ann Neurol 97:281–295 39487710 10.1002/ana.27116 PMC11740280

[129] Tan EL, Lope J, Bede P (2024) Harnessing big data in amyotrophic lateral sclerosis: machine learning applications for clinical practice and pharmaceutical trials. J Integr Neurosci 23:58 38538227 10.31083/j.jin2303058

[130] Tan HHG, Westeneng HJ, Nitert AD, van Veenhuijzen K, Meier JM, van der Burgh HK, van Zandvoort MJE, van Es MA, Veldink JH, van den Berg LH (2022) MRI clustering reveals three ALS subtypes with unique neurodegeneration patterns. Annals Neurol. 10.1002/ana.26488 10.1002/ana.26488 PMC9826424 36054734

[131] Bede P, Murad A, Lope J, Hardiman O, Chang KM (2022) Clusters of anatomical disease-burden patterns in ALS: a data-driven approach confirms radiological subtypes. J Neurol 10.1007/s00415-022-11081-3 PMC9294023 35333981

[132] Dukic S, McMackin R, Costello E, Metzger M, Buxo T, Fasano A, Chipika R, Pinto-Grau M, Schuster C, Hammond M, Heverin M, Coffey A, Broderick M, Iyer PM, Mohr K, Gavin B, McLaughlin R, Pender N, Bede P, Muthuraman M, van den Berg LH, Hardiman O, Nasseroleslami B (2022) Resting-state EEG reveals four subphenotypes of amyotrophic lateral sclerosis. Brain 145:621–631 34791079 10.1093/brain/awab322 PMC9014749

[133] Querin G, Bede P, Marchand-Pauvert V, Pradat PF (2018) Biomarkers of spinal and bulbar muscle atrophy (SBMA): a comprehensive review. Front Neurol 9:844 30364135 10.3389/fneur.2018.00844 PMC6191472

[134] Querin G, El Mendili MM, Lenglet T, Behin A, Stojkovic T, Salachas F, Devos D, Le Forestier N, Del Mar AM, Debs R, Lacomblez L, Meninger V, Bruneteau G, Cohen-Adad J, Lehericy S, Laforet P, Blancho S, Benali H, Catala M, Li M, Marchand-Pauvert V, Hogrel JY, Bede P, Pradat PF (2019) The spinal and cerebral profile of adult spinal-muscular atrophy: a multimodal imaging study. NeuroImage Clinical 21:101618 30522974 10.1016/j.nicl.2018.101618 PMC6413472

[135] Finegan E, Siah WF, Li Hi Shing S, Chipika RH, Hardiman O, Bede P (2022) Cerebellar degeneration in primary lateral sclerosis: an under-recognized facet of PLS. Amyotroph Later Scler Frontotemp Degener 1–12 10.1080/21678421.2021.2023188 34991421

[136] Finegan E, Kleinerova J, Hardiman O, Hutchinson S, Garcia-Gallardo A, Tan EL, Bede P (2025) Pseudobulbar affect: clinical associations, social impact and quality of life implications - Lessons from PLS. J Neurol 272:266 40072589 10.1007/s00415-025-12971-y PMC11903626

[137] Li Hi Shing S, Lope J, McKenna MC, Chipika RH, Hardiman O, Bede P (2021) Increased cerebral integrity metrics in poliomyelitis survivors: putative adaptation to longstanding lower motor neuron degeneration. J Neurol Sci 424:117361 33773768 10.1016/j.jns.2021.117361

[138] Li Hi Shing S, Lope J, Chipika RH, Hardiman O, Bede P (2021) Extra-motor manifestations in post-polio syndrome (PPS): fatigue, cognitive symptoms and radiological features. Neurol Sci 42:4569–4581 33635429 10.1007/s10072-021-05130-4

[139] Li Hi Shing S, Lope J, Chipika RH, Hardiman O, Bede P (2021) Imaging data indicate cerebral reorganisation in poliomyelitis survivors: Possible compensation for longstanding lower motor neuron pathology. Data Brief 38:107316 34485646 10.1016/j.dib.2021.107316 PMC8397913

[140] Turner MR, Gerhard A, Al-Chalabi A, Shaw CE, Hughes RAC, Banati RB, Brooks DJ, Leigh PN (2005) Mills’ and other isolated upper motor neurone syndromes: in vivo study with 11C-(R)-PK11195 PET. J Neurol Neurosurg Psychiatry 76:871–874 15897516 10.1136/jnnp.2004.047902 PMC1739672

[141] Bede P, Walsh R, Fagan AJ, Hardiman O (2013) “Sand-watch” spinal cord: a case of inferior cervical spinal cord atrophy. J Neurol. 10.1007/s00415-013-7193-7 24281771 10.1007/s00415-013-7193-7

[142] Sonwalkar HA, Shah RS, Khan FK, Gupta AK, Bodhey NK, Vottath S, Purkayastha S (2008) Imaging features in Hirayama disease. Neurol India 56:22–26 18310832 10.4103/0028-3886.39307

[143] Lebouteux MV, Franques J, Guillevin R, Delmont E, Lenglet T, Bede P, Desnuelle C, Pouget J, Pascal-Mousselard H, Pradat PF (2014) Revisiting the spectrum of lower motor neuron diseases with snake eyes appearance on magnetic resonance imaging. Eur J Neurol 21:1233–1241 24847978 10.1111/ene.12465

[144] Bede P, Finegan E (2018) Revisiting the pathoanatomy of pseudobulbar affect: mechanisms beyond corticobulbar dysfunction. Amyotroph Later Scler Frontotemp Degener 19:4–6 10.1080/21678421.2017.1392578 29092641

[145] Trojsi F, Di Nardo F, Santangelo G, Siciliano M, Femiano C, Passaniti C, Caiazzo G, Fratello M, Cirillo M, Monsurrò MR, Esposito F, Tedeschi G (2017) Resting state fMRI correlates of Theory of Mind impairment in amyotrophic lateral sclerosis. Cortex 97:1–16 29073458 10.1016/j.cortex.2017.09.016

[146] Turner MR, Barohn RJ, Corcia P, Fink JK, Harms MB, Kiernan MC, Ravits J, Silani V, Simmons Z, Statland J, van den Berg LH, Mitsumoto H (2020) Primary lateral sclerosis: consensus diagnostic criteria. J Neurol Neurosurg Psychiatry 91:373–377 32029539 10.1136/jnnp-2019-322541 PMC7147236

[147] Finegan E, Li Hi Shing S, Siah WF, Chipika RH, Chang KM, McKenna MC, Doherty MA, Hengeveld JC, Vajda A, Donaghy C, Hutchinson S, McLaughlin RL, Hardiman O, Bede P (2020) Evolving diagnostic criteria in primary lateral sclerosis: the clinical and radiological basis of “probable PLS.” J Neurol Sci 417:117052 32731060 10.1016/j.jns.2020.117052

[148] Brettschneider J, Del Tredici K, Toledo JB, Robinson JL, Irwin DJ, Grossman M, Suh E, Van Deerlin VM, Wood EM, Baek Y, Kwong L, Lee EB, Elman L, McCluskey L, Fang L, Feldengut S, Ludolph AC, Lee VM, Braak H, Trojanowski JQ (2013) Stages of pTDP-43 pathology in amyotrophic lateral sclerosis. Ann Neurol 74:20–38 23686809 10.1002/ana.23937 PMC3785076

[149] Müller HP, Del Tredici K, Lulé D, Müller K, Weishaupt JH, Ludolph AC, Kassubek J (2020) In vivo histopathological staging in C9orf72-associated ALS: a tract of interest DTI study. NeuroImage Clinical 27:102298 32505118 10.1016/j.nicl.2020.102298 PMC7270604

[150] Michielsen A, van Veenhuijzen K, Hiemstra F, Jansen IM, Kalkhoven B, Veldink JH, Kruitwagen ET, van Es M, van Zandvoort MJE, van den Berg LH, Westeneng HJ (2025) Cognitive impairment within and beyond the FTD spectrum in ALS: development of a complementary cognitive screen. J Neurol 272:268 40074931 10.1007/s00415-025-13006-2 PMC11903523

[151] Strong MJ, Abrahams S, Goldstein LH, Woolley S, McLaughlin P, Snowden J, Mioshi E, Roberts-South A, Benatar M, HortobaGyi T, Rosenfeld J, Silani V, Ince PG, Turner MR (2017) Amyotrophic lateral sclerosis - frontotemporal spectrum disorder (ALS-FTSD): Revised diagnostic criteria. Amyotroph Later Scler Frontotemp Degener 18:153–174 10.1080/21678421.2016.1267768 PMC7409990 28054827

[152] Roche JC, Rojas-Garcia R, Scott KM, Scotton W, Ellis CE, Burman R, Wijesekera L, Turner MR, Leigh PN, Shaw CE, Al-Chalabi A (2012) A proposed staging system for amyotrophic lateral sclerosis. Brain 135:847–852 22271664 10.1093/brain/awr351 PMC3286327

[153] Chiò A, Moglia C, Canosa A, Manera U, Vasta R, Brunetti M, Barberis M, Corrado L, D’Alfonso S, Bersano E, Sarnelli MF, Solara V, Zucchetti JP, Peotta L, Iazzolino B, Mazzini L, Mora G, Calvo A (2019) Cognitive impairment across ALS clinical stages in a population-based cohort. Neurology 93:e984–e994 31409738 10.1212/WNL.0000000000008063 PMC6745732

[154] Corcia P, Bede P, Pradat PF, Couratier P, Vucic S, de Carvalho M (2021) Split-hand and split-limb phenomena in amyotrophic lateral sclerosis: pathophysiology, electrophysiology and clinical manifestations. J Neurol Neurosurg Psychiatry. 10.1136/jnnp-2021-326266 34285065 10.1136/jnnp-2021-326266

[155] Eisen A, Turner MR, Lemon R (2014) Tools and talk: an evolutionary perspective on the functional deficits associated with amyotrophic lateral sclerosis. Muscle Nerve 49:469–477 24273101 10.1002/mus.24132

[156] Eisen A, Turner MR (2013) Does variation in neurodegenerative disease susceptibility and phenotype reflect cerebral differences at the network level? Amyotroph Later Scler Frontotemp Degener 14:487–493 10.3109/21678421.2013.812660 23879681

[157] Eisen A, Bede P (2021) The strength of corticomotoneuronal drive underlies ALS split phenotypes and reflects early upper motor neuron dysfunction. Brain Behav e2403 10.1002/brb3.2403 PMC8671797 34710283

[158] Eisen A, Braak H, Del Tredici K, Lemon R, Ludolph AC, Kiernan MC (2017) Cortical influences drive amyotrophic lateral sclerosis. J Neurol Neurosurg Psychiatry 88:917–924 28710326 10.1136/jnnp-2017-315573

[159] Henderson RD, Kepp KP, Eisen A (2022) ALS/FTD: evolution, aging, and cellular metabolic exhaustion. Front Neurol 13:890203 35711269 10.3389/fneur.2022.890203 PMC9196861

[160] Wolmer PS, de Borba FC, de Rezende TJR, González-Salazar C, Pedroso JL, Barsottini OGP, Kleinerova J, Bede P, Marques W Jr, França MC Jr (2025) Distinct patterns of cerebral and spinal pathology along the spectrum of ATXN2-related disorders. J Neurol 272:330 40204975 10.1007/s00415-025-13037-9

[161] Lulé DE, Müller HP, Finsel J, Weydt P, Knehr A, Winroth I, Andersen P, Weishaupt J, Uttner I, Kassubek J, Ludolph AC (2020) Deficits in verbal fluency in presymptomatic C9orf72 mutation gene carriers-a developmental disorder. J Neurol Neurosurg Psychiatry 91:1195–1200 32855285 10.1136/jnnp-2020-323671 PMC7569387

[162] Bede P, Siah WF, McKenna MC, Li Hi Shing S (2020) Consideration of C9orf72-associated ALS-FTD as a neurodevel-opmental disorder: insights from neuroimaging. J Neurol Neurosurg Psychiatry 91:1138 32855286 10.1136/jnnp-2020-324416

[163] Costello E, Rooney J, Pinto-Grau M, Burke T, Elamin M, Bede P, McMackin R, Dukic S, Vajda A, Heverin M, Hardiman O, Pender N (2021) Cognitive reserve in amyotrophic lateral sclerosis (ALS): a population-based longitudinal study. J Neurol Neurosurg Psychiatry. 10.1136/jnnp-2020-324992 33563807 10.1136/jnnp-2020-324992

[164] Temp AGM, Kasper E, Machts J, Vielhaber S, Teipel S, Hermann A, Prudlo J (2022) Cognitive reserve protects ALS-typical cognitive domains: a longitudinal study. Annals Clin Trans Neurol 9:1212–1223 10.1002/acn3.51623 PMC9380174 35866289

[165] Bede P, Bogdahn U, Lope J, Chang KM, Xirou S, Christidi F (2021) Degenerative and regenerative processes in amyotrophic lateral sclerosis: motor reserve, adaptation and putative compensatory changes. Neural Regen Res 16:1208–1209 33269779 10.4103/1673-5374.300440 PMC8224145

[166] Trojsi F, Di Nardo F, Caiazzo G, Siciliano M, D’Alvano G, Passaniti C, Russo A, Bonavita S, Cirillo M, Esposito F, Tedeschi G (2021) Between-sex variability of resting state functional brain networks in amyotrophic lateral sclerosis (ALS). J Neural Transm (Vienna) 128:1881–1897 34471976 10.1007/s00702-021-02413-0 PMC8571222

[167] Bede P, Elamin M, Byrne S, Hardiman O (2014) Sexual dimorphism in ALS: exploring gender-specific neuroimaging signatures. Amyotroph Later Scler Frontotemp Degener 15:235–243 10.3109/21678421.2013.865749 24344910

[168] Bede P, Iyer PM, Schuster C, Elamin M, McLaughlin RL, Kenna K, Hardiman O (2016) The selective anatomical vulnerability of ALS: ‘disease-defining’ and ‘disease-defying’ brain regions. Amyotroph Later Scler Frontotemp Degener 17:561–570 10.3109/21678421.2016.1173702 27087114

[169] Meier JM, van der Burgh HK, Nitert AD, Bede P, de Lange SC, Hardiman O, van den Berg LH, van den Heuvel MP (2020) Connectome-based propagation model in amyotrophic lateral sclerosis. Ann Neurol 87:725–738 32072667 10.1002/ana.25706 PMC7186838

[170] Eisen A, Lemon R, Kiernan MC, Hornberger M, Turner MR (2015) Does dysfunction of the mirror neuron system contribute to symptoms in amyotrophic lateral sclerosis? Clin Neurophysiol 126:1288–1294 25727900 10.1016/j.clinph.2015.02.003

[171] Goffin L, Lemoine D, Clotman F (2024) Potential contribution of spinal interneurons to the etiopathogenesis of amyotrophic lateral sclerosis. Front Neurosci 18:1434404 39091344 10.3389/fnins.2024.1434404 PMC11293063

[172] Castro J, Oliveira Santos M, Swash M, de Carvalho M (2024) Segmental motor neuron dysfunction in amyotrophic lateral sclerosis: Insights from H reflex paradigms. Muscle Nerve 69:303–312 38220221 10.1002/mus.28035

[173] Bede P (2017) Deciphering neurodegeneration: A paradigm shift from focality to connectivity. Neurology 89:1758–1759 28954879 10.1212/WNL.0000000000004582

[174] Bede P, Querin G, Pradat PF (2018) The changing landscape of motor neuron disease imaging: the transition from descriptive studies to precision clinical tools. Curr Opin Neurol 31:431–438 29750730 10.1097/WCO.0000000000000569

[175] Knight AC, Morrone CD, Varlow C, Yu WH, McQuade P, Vasdev N (2023) Head-to-head comparison of Tau-PET radioligands for imaging TDP-43 in post-mortem ALS brain. Mol Imag Biol 25:513–527 10.1007/s11307-022-01779-1 36258099

[176] Muller HP, Turner MR, Grosskreutz J, Abrahams S, Bede P, Govind V, Prudlo J, Ludolph AC, Filippi M, Kassubek J (2016) A large-scale multicentre cerebral diffusion tensor imaging study in amyotrophic lateral sclerosis. J Neurol Neurosurg Psychiatry 87:570–579 26746186 10.1136/jnnp-2015-311952

